# Thermal Desorption Used to Characterize Volatile Organic Compounds of Recycled Plastics

**DOI:** 10.3390/polym18070792

**Published:** 2026-03-25

**Authors:** Sandra Czaker, Joerg Fischer

**Affiliations:** 1 Institute of Polymeric Materials and Testing, Johannes Kepler University Linz, Altenbergerstraße 69, 4040 Linz, Austria; joerg.fischer@jku.at; 2 LIT Factory, Johannes Kepler University Linz, Altenbergerstraße 69, 4040 Linz, Austria

**Keywords:** volatile organic compounds, plastics recycling, thermal desorption, post-consumer material, contaminants, chromatography

## Abstract

About 10% of plastic products are recycled worldwide, highlighting the need for technology improvements based on deeper material understanding. In packaging, which holds the highest market share in plastics demand, odor and potential hazards remain critical barriers to high-quality recycling. Conventional characterization relies on chromatography with extensive sample preparation. A gas chromatography system equipped with thermal desorption and dual flame ionization and mass spectrometric detection (ATD-GC/FID-MS) was established to analyze recyclates directly, thereby accelerating technology adaptation and guiding follow-up analyses. For calibration and validation, liquid standards were introduced into TenaxTA-filled tubes via a packed column injector and compared to a loading rig. The injector exhibited losses for higher-molar-mass compounds and solvent-dependent signal shifts. A storage study on compounded recycled polypropylene stored under various conditions showed that samples not frozen in sealed containers should be analyzed within 30 days. Experiments with varying sample geometries demonstrated that higher surface-to-volume ratios increase volatile release and variability in results, highlighting the need for uniform shapes. Applying the method to recycled yogurt cups enables the identification and quantification of contaminants, facilitating optimization of the washing process. Overall, ATD-GC/FID-MS provides a rapid screening tool for recyclate quality control and supports the improvement of recycling technologies.

## 1. Introduction

High-quality plastics recycling still faces several barriers that need to be addressed. Considering the chemical cleanliness of the recyclates, which is critical for medical and food contact materials, product contaminants can cause unpleasant odors or pose health risks to consumers [1,2,3,4]. To overcome these barriers and increase recycling rates, technologies for pre-treatment and recyclate cleaning require improvements. This demands characterization methods to gather knowledge about the often fluctuating material inputs and the resulting recyclate properties.

Characterizing contaminants in post-consumer materials remains challenging. The enormous diversity of unknown compounds present in post-consumer material streams requires substantial effort to quantitatively extract and isolate them [5,6]. Identification and absolute quantification are often difficult because analytical standards are not available for every compound or degradation product formed during recycling. This knowledge is essential for predicting contaminant migration and assessing associated hazards [7]. Concerns that hazardous compounds may go undetected necessitate costly high-resolution instrumentation to achieve the required detection limits and specialized expertise. Consequently, it is unattractive to industry to apply such methods during technology development, which can hinder innovation towards high-quality recycling.

In case of contaminants in post-consumer plastics causing odor or potentially migrating from them into food or other media, screening for volatile organic compounds (VOCs) could cover a broad part of relevant contaminants [8]. VOCs are low to medium-molar-mass compounds with relatively high vapor pressures, allowing them to enter the gas phase easily. They are commonly monitored in the context of air quality, as well as emissions from furniture, building materials, coatings, and industrial sources [9,10,11]. The determination of VOC emissions from non-metallic car interiors is even specified in industrial standards [12,13]. This practice reflects the fact that airborne VOCs can pose a health risk and can cause odors when they interact with our olfactory cells. In recycled products, particularly packaging, they are of concern because their small size gives them greater mobility within the free volume of the polymer matrix, increasing the possibility of migration into contacting media [14,15,16,17].

For a screening of a variety of unknown compounds in solid samples of recycled plastics, a gas chromatograph (GC) combined with a mass spectrometer (MS) is commonly used [6]. Prior to the analysis, VOCs have to be extracted from the sample, typically via either headspace or solvent extraction techniques [18]. The latter employs various solvents and energy inputs to promote the transfer of VOC into a liquid phase and often involves multiple handling steps, increasing the risk of losses of the volatile analytes. In contrast, headspace techniques enable direct analysis of solid samples without solvents and frequently have the same or even more sensitivity to the recovery of VOCs.

In this study, a GC-MS method was developed utilizing an analytical thermal desorber (ATD) to thermally extract VOCs from solid samples. Besides the reduced time and resources regarding sample preparation, ATD uses a purge-and-trap system. By enriching analytes prior to GC injection, ATD provides improved sensitivity and lower detection limits, which is advantageous for contaminant screening and quantification. Compared to static and dynamic headspace techniques, where sampling of the equilibrated headspace is conducted, ATD typically achieves lower limits of detection. Moreover, quantification with ATD is often less labor-intensive because fewer extraction parameters need to be optimized [19]. Since the calibration standards were prepared in the liquid phase, they had to be transferred onto adsorbent-filled sample tubes before analysis. To accomplish this, two loading techniques were evaluated: loading with a packed column injector and loading with an external loading rig. Parameters like the sample storage and preparation of solid post-consumer material were also optimized. In a case study to test the applicability of the method, differently washed post-consumer polypropylene (PP) and polystyrene (PS) yogurt cups were analyzed. These cups originated from a separate collection of post-consumer waste, including thermoformed and injection-molded cups. The method revealed a high impact on cleanliness with cold washing and typical product-related contaminants.

Overall, this study aimed to develop an accessible method providing a screening tool to identify VOCs in recyclates, facilitating a fast adaptation of recycling technologies. It should also support the assessment of contaminants, thereby avoiding hasty decisions to conduct more expensive and time-consuming analyses from the start. Regular analysis of input waste streams and recycled products of an implemented recycling technology helps to improve the knowledge of the origin of contaminants and can ensure industrial recycling quality control. All of this could accelerate the required technology improvements aimed to ensure cleanliness of recycled polymers and may promote their circularity.

## 2. Experimental Work

The following method was developed to identify and quantify unknown volatile contaminants in plastic recyclates that may cause unpleasant odors or pose health risks. To evaluate contaminants in recyclates, a TurboMatrix 650 ATD (PerkinElmer Inc., Shelton, CT, USA) was coupled to a Clarus 690 GC (PerkinElmer Inc.). The built-in flame ionization detector (FID) and a Clarus SQ 8T MS (PerkinElmer Inc.) were linked via a post-column flow splitter to utilize both detectors simultaneously. Adjusting the relative lengths of the capillaries supplying the two detectors allowed recording of two chromatograms with synchronized retention times. This configuration provides qualitative data from the MS and more accurate quantitative data from the FID.

Given the wide variety of contaminants to be screened and the diverse types and compositions of plastics, a general-purpose separation column HP Ultra 2 size 50 m × ID 0.32 mm and 0.52 μm stationary phase (Agilent Technologies, Santa Clara, CA, USA) was installed. Separation of the contaminants was improved by optimizing the temperature program using a control mixture containing several nonpolar alkanes and polar compounds across a wide mass range, as listed in Table 1. Other system parameters are summarized in Table 2.

**Figure 1:**
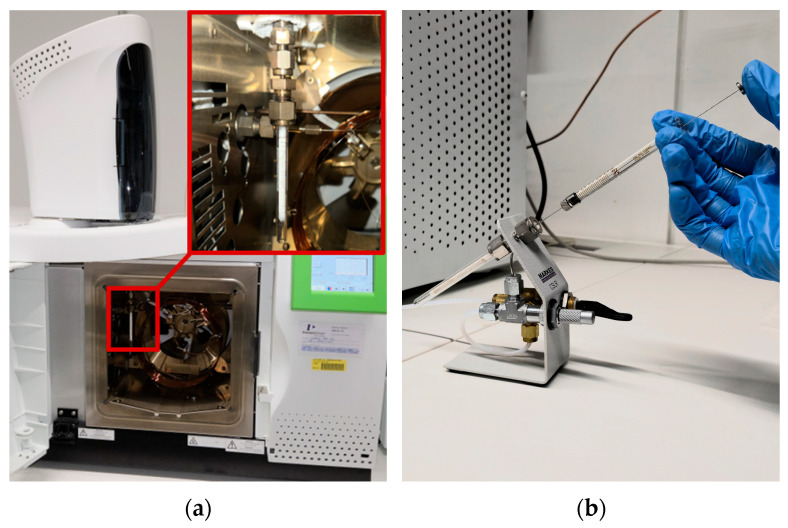
Pictures of the tube loading using (**a**) a packed column injector and (**b**) a calibration solution loading rig (CSLR).

Due to the high number of compounds expected in recyclates, concentrations were determined semi-quantitatively via a toluene standard of 0.5 mg/mL. This liquid standard solution had to be transferred to an adsorbent to be able to measure it with the ATD. For that purpose, a loading technique was required to transfer the standard solution to a sample tube filled with TenaxTA without losing the very volatile toluene. Two approaches were implemented:

- An adapted heated packed column injector operated in splitless mode, with the TenaxTA tube connected via an adapter, as shown in Figure 1a. This configuration allowed the use of the GC autosampler, ensuring consistent injection volumes under an inert carrier-gas flow without any additional device.
- Manual tube loading via a calibration solution loading rig (CSLR, Markes International, Bridgend, Wales, UK), enabling direct injection onto the TenaxTA under an inert gas flow, as shown in Figure 1b. This approach represents a low-temperature alternative to the heated packed column injector.

To compare the two loading techniques, volumes of 0.5, 1.0, 2.0, 3.0, and 4.0 µL of a toluene solution at 0.50 and 0.04 mg/mL were injected and analyzed. For the tube loading via the packed column injector, the injector was heated to 430 °C and the TenaxTA tube was conditioned in the GC oven to 30 °C. During autosampler injection, a constant helium flow of 20 mL/min transported the toluene to the TenaxTA and continued for 30 min to remove most of the methanol solvent. Manual loading via the CSLR was conducted at room temperature under a nitrogen flow of 100 mL/min for 6 min.

After choosing a loading technique, the influence of the sample storage on the resulting VOC values was assessed. For that purpose, a benchmark PP recyclate from pre-sorted post-consumer household waste (Dipolen PP, Borealis Group, Linz, Austria) was compounded to mimic the production step. The resulting granules were stored (i) open at room temperature in a 500 mL beaker, (ii) sealed at room temperature in an airtight aluminum bottle, and (iii) sealed at −32 °C in a headspace vial. With the ATD, it is possible to directly analyze the solid recyclate samples. However, the surface-to-volume ratio of the samples has a major impact on the results and must be considered for comparability [20]. Two sample shapes with markedly different surface-to-volume ratios, platelets and powder, were analyzed and compared. Platelets measuring 14 × 3 mm were punched from 1 mm-thick round plates, which were prepared using a heated laboratory press. Powder was produced by cryogenic milling, resulting in a particle size similar to table salt, though the size distribution was less uniform. The objective was to achieve a big difference in surface-to-volume ratios to highlight their significance. In both cases, the same post-consumer PP material was used. Subsequently, the differently stored granules and sample geometries were analyzed with the determined method parameters above.

Using the optimized method, a case study was finally conducted on differently washed post-consumer yogurt cup waste. The material input consisted of separately collected post-consumer yogurt cup waste, which was manually sorted into PP and PS, the predominant polymer types for this product [21]. After shredding, the resulting flakes were either (i) not washed, (ii) washed with water at 20 °C (cold), or (iii) washed with water heated to 40 °C (hot), and, for PP only, (iv) washed with water containing soda at 40 °C (hot + soda). Considering that the material originated from post-consumer yogurt cups, the goal was to close the loop and produce the same product again. To obtain a representative sample close to the final product, the pre-treated flakes were ground in a cutting mill and afterwards extruded into film sheets approximately 100 µm thick, matching the average wall thickness of deep-drawn yogurt cups. Before analyzing, the film sheets were cut into 30 × 10 mm pieces.

## 3. Results and Discussion

### 3.1. Influence of Calibration Solution Loading

**Figure 2:**
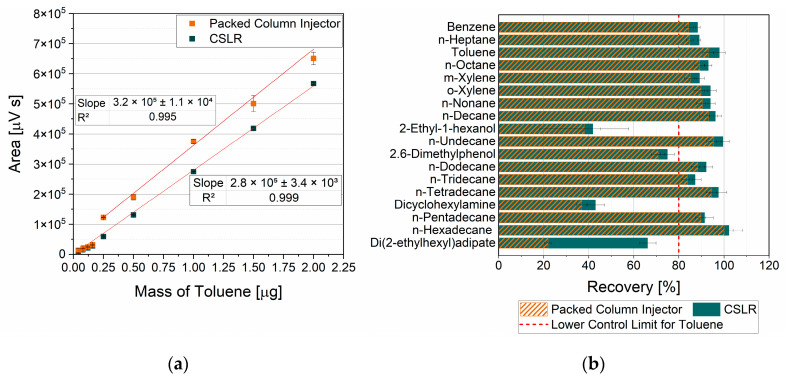
Validation results of tube loading techniques using a packed column injector and CSLR, including (**a**) calibration curves for toluene and (**b**) recoveries of a control mixture.

For the calibration, the standard solution needed to be transferred to a TenaxTA tube. The effect of loading with a packed column injector versus an external loading rig was compared. Calibration results are depicted in Figure 2a, exhibiting an excellent linear fit for the CSLR curve, explaining 99.9% of the variance (R^2^ = 0.999). Responses up to 0.16 µg toluene from the more diluted solution using the packed column injector were in good agreement with the CSLR responses. However, the more concentrated solution induced a jump to higher responses at 0.25 µg in the packed column injector curve. Fitting the datapoints from 0.25 mg and higher yielded a good linear fit, explaining 99.5% of the variance (R^2^ = 0.995), and a slightly steeper slope than the CSLR fit. This drift towards higher responses for the packed column injector indicates concentration dependence, potentially arising from a displacement mechanism in the injector driven by the large expansion volume of methanol. In this case, the methanol transiently occupied the TenaxTA and was subsequently purged off due to its low breakthrough volume. With increasing methanol volume, the available adsorption sites for toluene are temporarily reduced, causing partial blow-off.

Figure 2b shows the semi-quantitative recoveries of the compounds included in the control mixture. With both loading techniques, highly polar compounds exhibited significantly lower recoveries than nonpolar compounds. Responses were semi-quantitatively calibrated against toluene, which interacts favorably with the nonpolar stationary phase of the separation column. In contrast, polar compounds interact less ideally with this phase, leading to peak-shape distortion and result underestimation. The particularly low recovery of di(2-ethylhexyl)adipate with the packed column injector indicated that this less volatile compound remained in the injector. Follow-up measurements confirmed residual analyte carryover.

Overall, the CSLR loading technique yielded higher recoveries, consistent with the calibration curve drift observed for the packed column injector. Apart from that, the CSLR calibration curve exhibited high linearity. Accordingly, the limits of detection and quantification for toluene using the CSLR loading technique were determined according to DIN32645 to be 0.004 µg and 0.011 µg, respectively [22]. Based on these results and the relatively short loading time, subsequent analyses were calibrated using the CSLR loading technique.

### 3.2. Influence of Sample Storage

**Figure 3:**
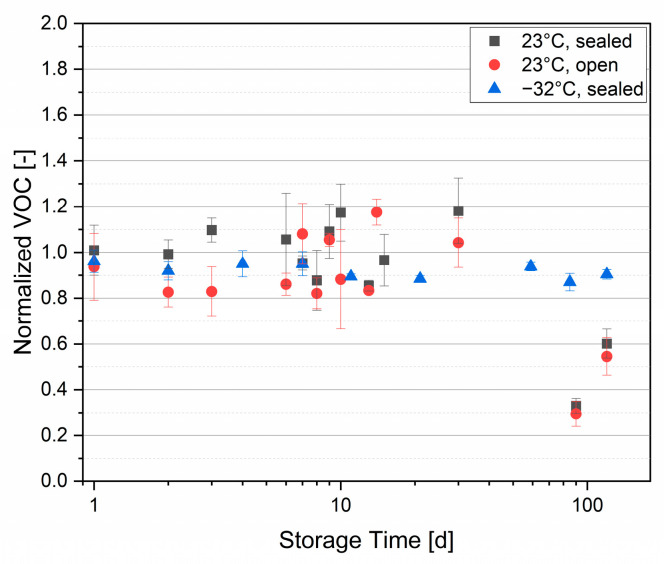
Normalized VOC emission over time of recycled polypropylene (PP) samples at various storage conditions.

VOC profiles are sensitive to time and temperature, which is critical when samples must be transported and stored prior to analysis. Even with short ways of transport, immediate measurement is not always feasible, making storage necessary. It is therefore important to understand how VOC emissions from recyclates change over time. Figure 3 shows substantial variability among replicates, reflecting the inherently inconsistent input stream of recyclates. Over the first 30 days, storage did not produce significant changes. By 90 days, emissions had decreased significantly for room temperature storage (*p* ≤ 7 × 10^−5^) according to a Welch two-sample t-test with 95% confidence interval, whereas frozen samples retained VOC levels comparable to the initial analysis. Based on these findings, samples should be measured within 30 days. For longer storage, samples are recommended to be stored in a deep freezer.

### 3.3. Influence of Sample Preparation

**Figure 4:**
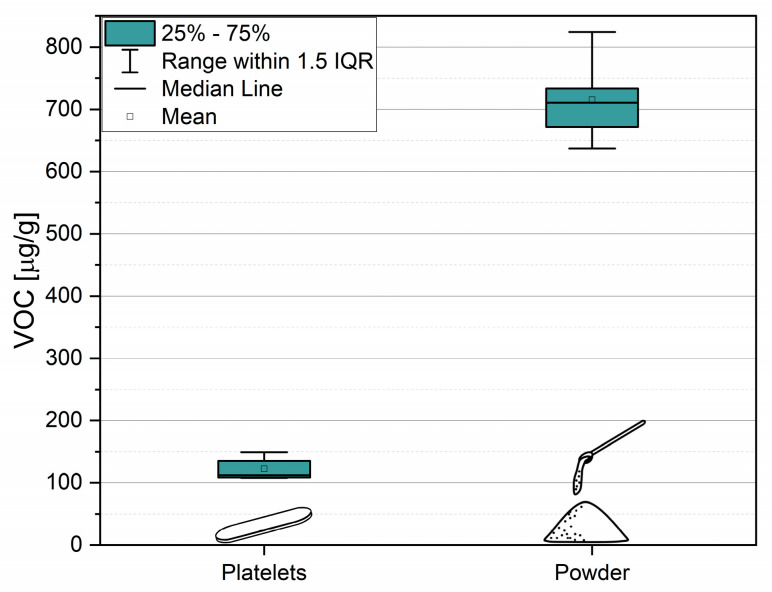
Statistical evaluation of VOC emission from the same recycled PP with different sample geometries.

The comparison of two sample geometries with markedly different surface-to-volume ratios and constant sample mass is shown in Figure 4. The powder samples exhibited approximately sixfold higher VOC emission compared to the platelets, but also a higher result variability. Consequently, the choice of sample geometry should reflect the analytical objective. A compact geometry that approximates the product shape is advantageous for estimating VOC emissions during use or storage. This can also be beneficial to make predictions about the migration potential of contaminants in packaging. In contrast, when the contaminants included in the bulk of the material have to be assessed, a high surface-to-volume ratio is required to maximize analyte release.

### 3.4. Case Study of Differently Washed Post-Consumer Yogurt Cup Material

**Figure 5:**
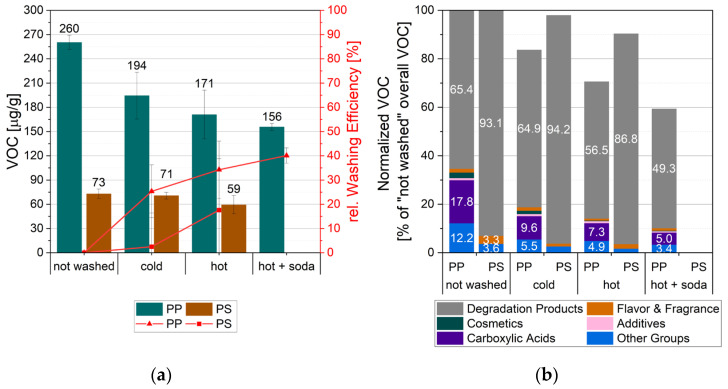
Effects of various washing conditions on (**a**) the overall VOC emission and relative washing efficiency, and (**b**) the VOC groups normalized to the overall VOC of the not-washed samples of post-consumer PP and polystyrene (PS).

The objective of this case study was to determine the washing efficiency and, more specifically, how the washing altered the contaminants in the product originating from this specific waste stream. Figure 5a shows the overall VOC values of the differently washed samples. The two polymer types show significant differences, with PP exhibiting more than threefold higher values than PS. For PP, cold washing removed approximately 25% of VOC, with modest additional improvement at elevated temperature and additional soda. In contrast, PS shows no significant change after cold washing, but hot washing results in a reduction of nearly 20% of VOC. On the one hand, the decrease in VOC values could result from a reduction in short-chain matrix molecules, potentially originating from degradation. On the other hand, it could result from the intended reduction in contaminants. The observed decreases may reflect both the intended removal of external contaminants and a reduction in short-chain matrix molecules.

According to Figure 5b, the findings clearly indicate that degradation products are the primary contributors to VOC emissions. Approximately 34% and 7% of the initial VOC values in not-washed PP and PS samples, respectively, were attributable to potential contaminants. These contaminants can be distinguished into contaminant groups, namely flavor & fragrance, cosmetics, additives, and carboxylic acids. “Other groups” comprised compounds that were not distinctly identified with the NIST Tandem Mass Spectral Library (Version 2.4, 2020, NIST Mass Spectrometry Data Center, Gaithersburg, MD, USA). This group could potentially be reduced with appropriate reference materials, which is challenging to select for recyclate mixtures. Comparing the two polymer types, the not-washed post-consumer PS is substantially less contaminated than the not-washed post-consumer PP, which is consistent with their thermorheological behavior at application temperatures. While PS is in the glassy state with low free volume, PP is in a rubbery state with more free volume, promoting contaminant sorption and migration [23].

Relative to not-washed samples, the values of all VOC groups in PP decrease significantly with increasing temperature and additional soda, supporting the interpretation that overall VOC reduction reflects material decontamination. In PP samples, several carboxylic acids were found in relatively high concentrations. These may originate from photo-oxidative degradation of polyolefins. Two contaminants classified under cosmetics were also carboxylic acids, and could alternatively be assigned to the flavor and fragrance category. For PS, cold washing affected the contaminant groups despite minimal changes in the overall VOC values. Hot washing of PS demonstrated a comparable decontamination effect to cold washing, while decreasing the degradation products by approximately 7%. Qualitatively, PS showed few contaminants primarily associated with flavoring and fragrance.

Eventually, contaminants in both materials were mainly reduced by cold washing. Depending on the application of the recyclates, these findings may help to select the most efficient pre-treatment procedure. Food-contact materials with high purity requirements, such as yogurt cups, require a detailed evaluation of each detected substance. However, none of the contaminants was classified as mutagenic, carcinogenic, or toxic to reproduction according to online databases [24,25]. Given this profile, migration testing may be recommended, particularly for the comparatively clean PS material. In general, the separate collection of yogurt cups led to contaminants typical for this product, but of low concern.

## 4. Conclusions

With the developed method, including an analytical thermal desorber coupled to a gas chromatograph with a mass spectrometer, volatile organic contaminants from solid plastic recyclate samples can be determined and quantified. Preferably, the calibration standard solution should be transferred to TenaxTA-filled sample tubes via an external loading rig to avoid the concentration-dependent calibration drift and losses with higher-molar-mass compounds that occurred using the packed column injector. It is also recommended to analyze recyclate samples within 30 days when stored at 23 °C or freeze the sealed samples below −32 °C to prolong storage time if required. To ensure comparability, the samples should be prepared with a uniform sample geometry, as powder samples showed sixfold higher emissions but also more result variability compared to platelets. Their surface-to-volume ratio should be either similar to the intended product for results close to reality or as high as possible to maximize contaminants release.

This method provides rapid and solvent-free sample preparation with minimal extraction steps, preventing losses of analytes. It enables direct semi-quantitative and qualitative assessment of contaminants causing unwanted odor in or migration from solid recyclates, as shown in the case study. Reliable results were obtained by calibrating with an external loading rig and using samples with uniform geometry, ensuring they were not frozen for more than 90 days or stored at room temperature for over 30 days. Analysis of film sheets produced from separately collected yogurt cups revealed that polystyrene cups are five times less contaminated than polypropylene cups, making them appealing for recycling into food-contact materials. In contrast, around one-third of the determined VOC values in the polypropylene samples were identified as contaminants, which were reduced by around 14% via washing of the input flakes. The method offers practical guidance to improve industrial cleaning processes with minimal energy consumption. This method can act as a preliminary test to decide whether more costly and time-consuming (regulatory) analyses are already appropriate to pursue. Finally, the information offered with this method contributes to the technology improvement required to bring plastics into circularity.

## Figures and Tables

**Table 1:** List of compounds included in the control mixture.

Compound	Molar Mass [g/mol]	Polarity
Benzene	78.1	nonpolar
n-Heptane	100.2	nonpolar
Toluene	92.1	nonpolar
n-Octane	114.2	nonpolar
m-Xylene	106.2	nonpolar
o-Xylene	106.2	nonpolar
n-Nonane	128.3	nonpolar
n-Decane	142.3	nonpolar
2-Ethyl-1-hexanol	130.2	polar
n-Undecane	156.3	nonpolar
2.6-Dimethylphenol	122.2	polar
n-Dodecane	170.3	nonpolar
n-Tridecane	184.4	nonpolar
n-Tetradecane	198.4	nonpolar
Dicyclohexylamine	181.3	polar
n-Pentadecane	212.4	nonpolar
n-Hexadecane	226.4	nonpolar
Di(2-ethylhexyl)adipate	370.6	polar

**Table 2:** Analysis Parameters of the analytical thermal desorber (ATD), gas chromatograph (GC), flame ionization detector (FID), and mass spectrometer (MS).

ATD	
Mode	two-stage desorption
Carrier Gas	He 5.0
Column Head Pressure [kPa]	130
Desorption Flow [mL/min]	40
Inlet Split Flow [mL/min]	44
Outlet Split Flow [mL/min]	19
Trap Temperature [°C]	−30 to 280
Heating Rate [°C/s]	99
Trap Hold [min]	20
Valve Temperature [°C]	280
Transfer Line Temperature [°C]	290
Desorption Temperature [°C]	90
Desorption Time [min]	30
**GC**	
Oven Program:	
Start Temperature	60 °C, 13 min
Ramp 1	6 °C/min to 215 °C
Ramp 2	25 °C/min to 280 °C
End Temperature	280 °C, 12 min
**FID**	
FID Temp. [°C]	300
Hydrogen Flow [mL/min]	30
Synthetic Air Flow [mL/min]	450
**MS**	
Ionization Mode	EI
Ion Source Temperature [°C]	250
Transfer Line Temperature [°C]	280
MS Mode	Scan Mode
Mass Range [m/z]	29–450
MS Solvent Delay [min]	4.5

## Data Availability

The original contributions presented in this study are included in the article. Further inquiries can be directed to the corresponding author.

## Abbreviations

The following abbreviations are used in this manuscript:

ATD-GC/FID-MS	Analytical thermal desorber coupled to a gas chromatograph including a flame ionization detector and connected to a mass spectrometer
VOC	Volatile organic compounds
GC	Gas chromatograph
MS	Mass spectrometer
ATD	Analytical thermal desorber
PP	Polypropylene
PS	Polystyrene
FID	Flame ionization detector
CSLR	Calibration solution loading rig
